# Hair Manganese as a Marker of Cardiometabolic Status Rather than Coronary Artery Disease Severity—An Exploratory Pilot Study

**DOI:** 10.3390/nu18071089

**Published:** 2026-03-28

**Authors:** Ewelina A. Dziedzic, Aleksandra Czernicka, Agnieszka Mazur-Jax, Andrzej Osiecki, Jakub S. Gąsior, Jakub Marek Baran, Łukasz Dudek, Wacław Kochman

**Affiliations:** 1 Cardiovascular Clinic, Centre of Postgraduate Medical Education (CMKP), 01-813 Warsaw, Poland; aleksandra.czernicka1203@gmail.com (A.C.); uskamazur@gmail.com (A.M.-J.); w.kochman@icloud.com (W.K.); 2 Department of Cardiology, Bielański Hospital, 01-809 Warsaw, Poland; mcosiek@gmail.com (A.O.); jak.baran00@gmail.com (J.M.B.); dudeklukasz96@gmail.com (Ł.D.); 3 Department of Pediatric Cardiology and General Pediatrics, Medical University of Warsaw, 02-091 Warsaw, Poland; jakub.gasior@wum.edu.pl

**Keywords:** manganese, trace elements, acute coronary syndrome, coronary artery disease

## Abstract

Background: Manganese (Mn) is an essential trace element with antioxidant properties; however, excessive exposure may contribute to inflammation and vascular dysfunction. Hair analysis provides an indicator of long-term Mn exposure. This study evaluated the relationship between hair Mn levels, acute coronary syndrome (ACS), coronary artery disease (CAD) severity, and cardiovascular risk factors, with particular emphasis on metabolic status in a cardiometabolic population. Methods: Hair Mn concentration was measured using inductively coupled plasma optical emission spectrometry (ICP-OES) in 80 patients (mean age 67 ± 11 years; 28.8% women) undergoing coronary angiography for suspected ACS. Final diagnoses included stable CAD (N = 42) and ACS [ST-elevation myocardial infarction (STEMI) N = 17, non-ST-elevation myocardial infarction (NSTEMI) N = 12, and unstable angina (UA) N = 9]. CAD severity was quantified using the SYNTAX score and the Coronary Artery Surgery Study Score (CASSS). Associations with clinical variables were assessed using non-parametric tests and Spearman correlations. The median SYNTAX score was 13.8 (range 0.0–68.5), and the median hair Mn concentration was 0.22 ppm (range 0.01–1.65). Results: SYNTAX scores were higher in ACS than in stable CAD (*p* = 0.027), with the highest values observed in NSTEMI. Hair Mn levels did not differ among diagnostic groups and showed no association with CASSS or SYNTAX (R = −0.11; *p* = 0.348). No differences were detected with respect to sex, smoking, prior myocardial infarction, hypertension, hyperlipidemia, or type 2 diabetes. A modest inverse correlation was observed between hair Mn and body mass index (BMI) in unadjusted analysis (R = −0.25; *p* = 0.03), but this association was not robust after correction for multiple comparisons, suggesting a potential exploratory link between manganese homeostasis and cardiometabolic status. Conclusions: Although hair Mn concentration was not associated with angiographic indices of CAD severity or ACS subtypes, the observed relationship with BMI may indicate a role of Mn homeostasis in cardiometabolic regulation. Larger prospective studies are required to clarify these associations.

## 1. Introduction

Cardiovascular diseases (CVDs) remain the leading cause of death worldwide, accounting for approximately 17.9 million deaths annually, of which around 85% are attributed to ischemic heart disease (including coronary artery disease, CAD) and stroke [1,2]. The development of CAD can be substantially modified through the control of major risk factors, such as dyslipidemia, diabetes, and smoking [3,4]. These factors contribute to the development of atherosclerosis—a chronic inflammatory process within the arterial wall initiated by lipoprotein retention [5]. This process involves inflammatory activation and contributes to plaque instability [6]. These processes may be modulated by trace elements, including manganese (Mn), an essential bioelement involved in numerous metabolic processes [7].

The main sources of manganese include diet, drinking water, and air [8]. Following intestinal absorption, Mn is distributed to various tissues, where it contributes to redox homeostasis [9]. A particularly important role in the pathogenesis of atherosclerosis is played by manganese-dependent mitochondrial superoxide dismutase (MnSOD), which neutralizes reactive oxygen species (ROS) and reduces oxidative stress—one of the key mechanisms underlying endothelial dysfunction and plaque progression [9,10,11,12,13,14,15].

Manganese exhibits a biphasic effect on the cardiovascular system. At physiological concentrations, it supports antioxidant defense mechanisms and maintains redox homeostasis [9,10,11], whereas chronic excessive exposure may lead to toxic accumulation in tissues [10,16,17,18,19,20,21,22,23]. Excess Mn may increase mitochondrial ROS production and activate inflammatory pathways, thereby contributing to endothelial dysfunction and atherosclerosis progression [19,20,21,22]. Given its narrow range between physiological requirement and toxicity, manganese represents a trace element linking metabolic regulation and cardiovascular disease [10,16,17,18,19,20,21,22,23].

Another challenge in evaluating the role of manganese in cardiovascular diseases is the selection of an appropriate method for assessing its concentration. Blood and urine analyses are most commonly used; however, their diagnostic value is limited due to short-term fluctuations in manganese levels [24]. Hair analysis is used as a biomarker of long-term exposure, as it reflects cumulative exposure [18,19,25,26]. Manganese concentrations in hair are typically higher than in blood or urine, facilitating detection [27]. Additional advantages of this method include its non-invasive nature and ease of storage and transport [28,29].

Although several studies have investigated the association between manganese and cardiovascular outcomes, most analyses have been based on blood or urine measurements or dietary intake assessments [24,30,31,32,33,34,35]. Data on long-term exposure biomarkers, such as manganese measured in hair in patients with CAD, remain limited [18,19,25,26]. These findings require further validation.

In clinical studies, the severity of coronary atherosclerosis is most commonly assessed using coronary angiography with validated anatomical scoring systems. One of the most widely used methods is the SYNTAX score, which serves as an independent predictor of cardiovascular events [36,37]. Additionally, the Coronary Artery Surgery Study Score (CASSS) is used to classify CAD severity based on the number of significantly stenosed major coronary vessels [38].

To our knowledge, studies evaluating hair manganese concentrations in patients with both chronic and acute coronary syndromes remain scarce. We hypothesized that long-term manganese exposure, reflected by its concentration in hair, may be associated with both angiographically assessed severity of CAD and selected cardiometabolic risk factors.

The aim of this study was to evaluate the role of manganese in coronary artery disease by: (a) comparing hair Mn concentrations in patients with chronic coronary syndrome (CCS) and acute coronary syndrome (ACS); (b) assessing the association between manganese levels and the severity of atherosclerosis evaluated using the Coronary Artery Surgery Study Score (CASSS) and the SYNTAX score; and (c) evaluating the relationship between manganese levels and classical cardiovascular risk factors, including age, sex, hypertension, type 2 diabetes, body mass index (BMI), smoking status, and lipid profile.

## 2. Materials and Methods

### 2.1. Experimental Approach

This retrospective observational study evaluated the relationship between long-term manganese (Mn) exposure and cardiovascular status in patients undergoing coronary angiography for suspected acute coronary syndrome (ACS). The experimental design comprised several sequential steps. First, eligible patients were selected from a hospital cohort based on predefined inclusion and exclusion criteria to minimize confounding factors related to external Mn contamination or severe comorbidities. Second, demographic characteristics, cardiovascular risk factors, anthropometric parameters, and routine laboratory results were collected from clinical records obtained during hospitalization. Third, the severity of coronary artery disease (CAD) was assessed using coronary angiography and quantified with established scoring systems. Fourth, hair samples were collected from the occipital region of the scalp and analyzed for Mn concentration using inductively coupled plasma optical emission spectrometry (ICP-OES), providing an indicator of long-term exposure. Finally, statistical analyses were performed to examine associations between hair Mn levels, CAD severity indices, ACS subtypes, and cardiometabolic risk factors. This study was designed as an exploratory observational study. Although no formal a priori sample size calculation was performed, an a posteriori estimation based on power analysis principles was provided (see Section 2.7). The sample consisted of consecutively enrolled eligible patients with complete clinical and laboratory data during the study period.

### 2.2. Study Population and Inclusion Criteria

Eligibility required age ≥ 18 years, hospitalization with clinical suspicion of ACS necessitating diagnostic coronary angiography, availability of complete clinical and laboratory documentation, and collection of a hair sample for trace element analysis.

This study included 80 patients (23 women and 57 men) admitted to the Cardiology Department of Bielański Hospital in Warsaw, Poland, between 2013 and 2017, who underwent coronary angiography for suspected acute coronary syndrome (ACS).

To minimize confounding, only participants without occupational manganese exposure or supplementation were included, and all were residents of Warsaw. Potential confounding factors such as environmental exposure and lifestyle characteristics were considered during patient selection, although not directly quantified. Environmental sources of Mn were not directly measured and may therefore represent residual confounding.

Patients in whom myocardial infarction was ruled out were assigned to the stable CAD comparison group.

The study was approved by the Bioethics Committee of the Medical University of Warsaw (approval number KB/124/2014) and conducted in accordance with the Declaration of Helsinki. Written informed consent was obtained from all participants.

### 2.3. Exclusion Criteria

Participants who dyed their hair or had wavy hair longer than 3 cm were excluded. Additional exclusion criteria included active malignancy, ongoing viral or bacterial infection, chronic kidney disease stages III–V, elevated inflammatory markers, terminal illness, and the use of manganese-containing supplements. Individuals with a history of thrombosis or vascular restenosis were also excluded.

### 2.4. Laboratory Test and Clinical Data

Venous blood samples were collected on admission and processed within two hours. Routine laboratory analyses included complete blood count, plasma glucose concentration, and serum lipid profile, comprising total cholesterol (TC), low-density lipoprotein cholesterol (LDL), high-density lipoprotein cholesterol (HDL), and triglycerides (TG). The diagnosis of type 2 diabetes mellitus or prediabetes was established following the 2019 ESC Guidelines on diabetes, prediabetes, and cardiovascular disease. Diagnostic criteria comprised fasting plasma glucose levels exceeding 126 mg/dL (≥7.0 mmol/L) confirmed on two separate occasions, the presence of clinical symptoms of diabetes accompanied by a random plasma glucose concentration of ≥200 mg/dL (≥11.1 mmol/L), or a plasma glucose concentration of ≥200 mg/dL (≥11.1 mmol/L) measured 120 min after an oral glucose tolerance test (OGTT) [39]. Hyperlipidemia was defined in accordance with the 2019 ESC/EAS Guidelines for the management of dyslipidemias as a lipid profile not achieving therapeutic targets specified for the individual’s cardiovascular risk category [40]. Hypertension was diagnosed when blood pressure measurements exceeded 140/90 mmHg, in line with the 2024 European Society of Hypertension practice guidelines [41]. Nutritional status was assessed using the body mass index (BMI), calculated as body weight in kilograms divided by the square of height in meters (kg/m^2^). Obesity was classified as a BMI of 30 kg/m^2^ or greater [42].

### 2.5. Hair Sample Analysis

Hair samples (0.2–0.3 g) were collected during hospitalization, prior to or shortly after coronary angiography, from multiple sites on the occipital scalp near the root, ensuring they were undyed. Samples were washed in a 1:100 solution of Triton X-100 (Sigma Aldrich Sp. z.o.o., Poznań, Poland) in an ultrasonic bath for 5 min, rinsed sequentially with high-purity water, acetone, and water, and dried to constant weight. A 0.15 g portion of each dried sample was digested in 4 mL of 65% nitric acid and 1 mL of 30% hydrogen peroxide (Merck, Darmstadt, Germany) in an 8 mL polypropylene tube, followed by incubation at 80 °C for 30 min in a microwave station. After cooling, the digested samples were diluted to 10 mL with Milli-Q water and analyzed for manganese using inductively coupled plasma optical emission spectrometry (ICP-OES; iCAP7400, Thermo Scientific, Waltham, MA, USA). Certified standards (CGZN1 and CGCU1, Inorganic Ventures, Christiansburg, VA, USA) were used for calibration and to verify elemental concentrations. Hair Mn concentrations in the present study were within the range commonly reported in general populations; however, the absence of standardized reference values complicates the interpretation of biomonitoring data. The World Health Organization (WHO) and other international agencies highlight the importance of monitoring manganese exposure due to its dual role as an essential nutrient and a potential environmental toxin.

### 2.6. Coronary Angiography

All participants underwent coronary angiography through either radial or femoral access to evaluate the extent of coronary artery stenosis. When clinically indicated, patients were referred for revascularization, with percutaneous coronary intervention (PCI) as the preferred approach [43]. The diagnosis of acute coronary syndrome (ACS) was established following the European Society of Cardiology guidelines. According to these criteria, ACS is diagnosed when there is an elevation in a biomarker of myocardial injury—specifically cardiac troponin—with at least one measurement exceeding the 99th percentile of the upper reference limit, together with at least one of the following: clinical manifestations of myocardial ischemia, new ischemic alterations on the electrocardiogram (ECG), appearance of pathological Q waves on the ECG, imaging evidence of myocardial viability loss, newly developed regional wall motion abnormalities consistent with ischemia, or angiographic visualization of an intracoronary thrombus [44]. Coronary atherosclerosis severity was evaluated using the SYNTAX scale and the Coronary Artery Surgery Study Score (CASSS). The SYNTAX score incorporates multiple factors, including the number, location and hemodynamic significance of coronary lesions. It serves as an independent predictor of long-term adverse cardiovascular events and informs the selection of revascularization strategies, the procedural approach and postoperative management [36,37]. Coronary angiography was conducted in all study participants using standard diagnostic catheters via either radial or femoral artery access. The extent of coronary atherosclerosis was evaluated according to the Coronary Artery Surgery Study Score (CASSS). A stenosis exceeding 70% in any of the major epicardial coronary arteries—the left anterior descending (LAD), left circumflex (LCX), or right coronary artery (RCA)—was assigned 1 point. In the case of left main coronary artery narrowing ≥50%, 2 points were given, corresponding to a two-vessel disease classification. The total CASSS value represents the sum of points from all affected vessels, allowing categorization into single-, double-, or triple-vessel coronary artery disease [38].

### 2.7. Statistical Analysis

The sample size was estimated based on power analysis for Pearson’s correlation coefficient. Assuming a moderate effect size, a two-tailed significance level of α = 0.05, and a statistical power of 80% (1 − β = 0.80), a minimum of approximately 82–85 participants is required to detect a moderate correlation with 80% power. The final sample size (N = 80) was therefore slightly below this threshold, which is acknowledged as a limitation.

Normality of data distribution was assessed using the Shapiro–Wilk test. As most continuous variables showed non-normal distribution, they are presented as median (interquartile range), whereas categorical variables are expressed as number (percentage). Categorical variables were compared using Pearson’s chi-square test or Fisher’s exact test, as appropriate. Differences between two independent groups for continuous variables were assessed using the Mann–Whitney U test. For comparisons involving more than two groups, the Kruskal–Wallis test was applied, followed by Dunn’s multiple-comparisons post hoc test. Correlations between hair manganese concentration and selected clinical or laboratory parameters were analyzed using the Spearman rank correlation coefficient. To improve interpretation of these exploratory analyses, 95% confidence intervals were calculated for correlation coefficients. In view of multiple parallel comparisons, *p*-values were additionally adjusted using the Benjamini–Hochberg false discovery rate method, with Bonferroni correction used as a conservative sensitivity approach. To assess whether manganese concentration was independently associated with coronary artery disease severity, multivariable linear regression was performed with CASSS as the dependent variable. To examine its relationship with clinical presentation, multivariable logistic regression was performed with unstable coronary artery disease as the dependent variable. Both models were adjusted for clinically relevant covariates, including age, sex, diabetes status, smoking, hypertension, LDL cholesterol, and BMI. Results of regression analyses are reported as beta coefficients or odds ratios, together with 95% confidence intervals and *p*-values. All statistical tests were two-sided, and a *p*-value of <0.05 was considered statistically significant. Statistical analyses were performed using Statistica 13 (StatSoft Inc., Tulsa, OK, USA), while supplementary analyses related to confidence intervals, correction for multiple testing, and multivariable modeling were conducted in Python 3.

## 3. Results

### 3.1. Study Population

The study included 80 participants (N = 23, 28.75% females) with a mean age of 67 ± 11 years. The median BMI value was 27 kg/m^2^ (range: 17–45). A total of 26 (32.50%) participants had a normal body weight, 35 (43.75%) were overweight, and 19 (23.75%) patients were classified as obese. Active smoking during the study was declared by 22 (27.50%) patients, and 7 (8.75%) patients had smoked in the past. Hypertension was present in 70 (87.50%) patients. A history of type 2 diabetes mellitus (t2DM) or diagnosis during the current hospitalization was found in 25 (31.25%) patients, and pre-diabetes in 6 (7.5%) patients. On the basis of the lipid profile (total cholesterol—TC, LDL and HDL cholesterol, triglycerides—TG), hyperlipidemia was assessed in 74 patients and diagnosed in 33 (41.25%). A history of myocardial infarction (MI) was noted in 26 (32.50%) patients. Acute coronary syndrome (ACS) as the cause of hospitalization was diagnosed in 38 (47.50%) patients (STEMI N = 17, 21.25%; NSTEMI N = 12, 15.00%; UA N = 9, 11.25%), while stable CAD was the cause in 42 (52.50%) patients. Insignificant changes in the coronary arteries (CASSS 0) were found in 15 (18.75%) patients. One-vessel coronary disease (CASSS 1) was found in 21 (26.25%) patients, two-vessel (CASSS 2) in 26 (32.50%), and three-vessel (CASSS 3) in 18 (22.50%) patients. The median SYNTAX score and Mn concentration were 13.8 points (range: 0.0–68.5) and 0.22 parts per million (ppm) (range: 0.01–1.65).

### 3.2. Differences in Mn Level Between Patients with Different Diagnoses

Mn concentration was evaluated in relation to demographic, metabolic, and angiographic variables, with particular focus on coronary artery disease severity. Table 1 presents the results for patients with different diagnoses: stable CAD, STEMI, NSTEMI, and UA in measured parameters. Significant differences were found in the distribution of patients with total cholesterol and LDL cholesterol. Additionally, there was a significant difference in SYNTAX score between patients with stable CAD and ACS. Patients with NSTEMI exhibited the highest SYNTAX score. However, no significant differences in manganese concentration were observed among patients with different diagnoses.

Significant differences were observed in the distribution of sex among CASSS groups. Additionally, there were notable differences in the distribution of patients with different CAD advancement and a history of previous myocardial infarction and differences in SYNTAX score between patients with different CAD advancement. There was no significant correlation between Mn levels and the advancement of CAD (Table 2).

**Figure 1:**
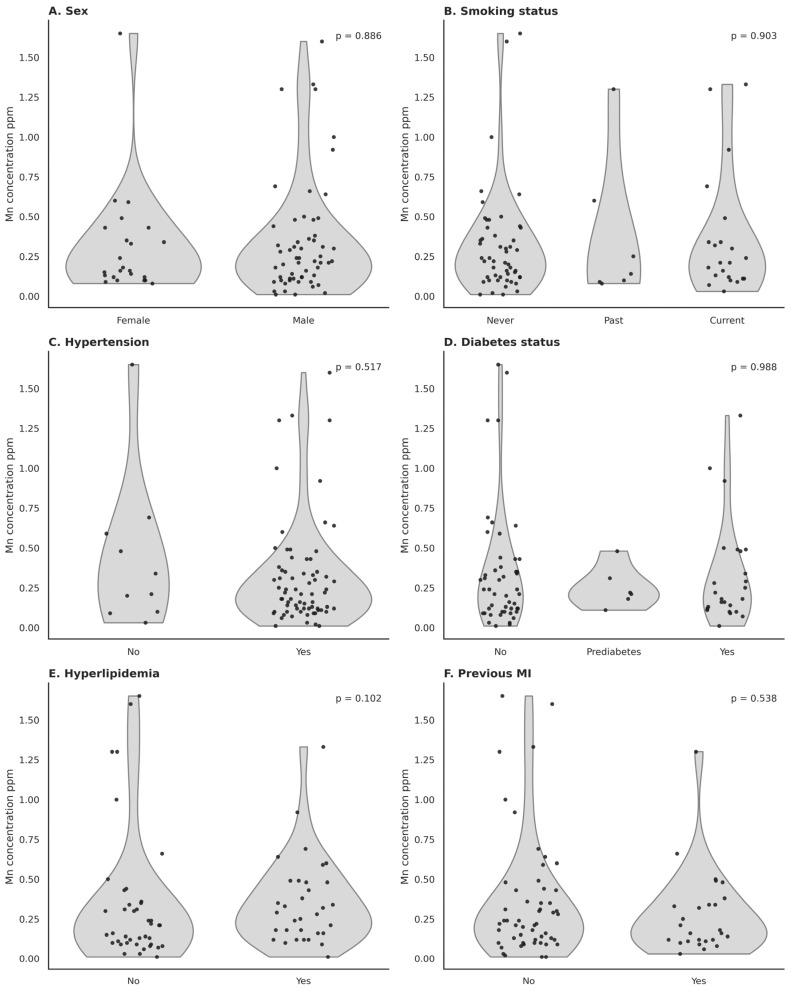
Association between Mn Level and Selected Parameters: Sex, Smoking Status, Hypertension, Type 2 Diabetes Mellitus (T2DM), Hyperlipidemia, and Previous Myocardial Infarction (MI).

No significant differences were found in Mn levels between males and females (Figure 1A). Similarly, there were no significant differences in Mn levels among participants with different smoking statuses (Figure 1B) or between patients with or without a history of previous myocardial infarction (MI) (Figure 1F). Furthermore, there was no significant correlation observed between Mn levels and patients with or without hypertension (Figure 1C), hyperlipidemia (Figure 1E), or type 2 diabetes (Figure 1D).

A nominal inverse association was observed between Mn concentration and BMI. No significant correlation was observed between Mn levels and age, lipid profile, or SYNTAX score (Table 3).

In the unadjusted analyses, Mn concentration was not significantly associated with CASSS, indicating no clear monotonic relationship between Mn level and angiographic coronary atherosclerosis. Similarly, no significant associations were observed between Mn concentration and age, HDL cholesterol, LDL cholesterol, TG, or SYNTAX score. The correlation estimates were generally small, and the corresponding 95% CI crossed zero, supporting the absence of a robust association.

Among the evaluated continuous variables, the only nominally significant finding in the unadjusted analysis was an inverse correlation between Mn concentration and BMI. This association was weak in magnitude, with a Spearman correlation coefficient of −0.250 and a 95% CI from −0.450 to −0.026. However, after adjustment for multiple comparisons using the Benjamini–Hochberg procedure or Bonferroni correction, this association was no longer statistically significant.

To further account for potential confounding, multivariable regression analyses were performed. In the adjusted linear regression model, Mn concentration was not independently associated with CASSS after controlling for age, sex, diabetes status, smoking, hypertension, LDL cholesterol, and BMI (beta = −0.323, 95% CI −1.015 to 0.369, *p* = 0.354). In this model, hypertension was the only variable independently associated with higher CASSS (beta = 0.804, 95% CI 0.020 to 1.588, *p* = 0.045). Likewise, in the adjusted logistic regression model, Mn concentration was not independently associated with ACS (OR = 0.282, 95% CI 0.049 to 1.606, *p* = 0.154), whereas LDL cholesterol remained significantly associated with ACS (OR = 1.024, 95% CI 1.007 to 1.040, *p* = 0.005). These findings indicate that Mn concentration was not an independent predictor of angiographic CAD severity or ACS in the studied group. Although isolated weak associations were observed in unadjusted analyses, they were not robust after correction for multiple testing and adjustment for relevant clinical covariates.

## 4. Discussion

In our study, no significant association was found between manganese concentrations in hair and the severity of coronary atherosclerosis assessed by the CASSS and SYNTAX scores, nor with the diagnosis of acute coronary syndrome (ACS). These findings suggest that long-term manganese status, as reflected by hair concentrations, does not directly correspond to either the angiographic extent of coronary artery disease or its acute clinical presentation. The use of hair as a biological matrix enables assessment of long-term manganese exposure, in contrast to serum measurements or dietary data, which primarily reflect short-term fluctuations. Studies evaluating manganese status in patients with coronary artery disease using hair remain limited, and most available evidence is based on serum analyses or dietary intake assessments [30,31,32,45]. This distinction is important when interpreting the biological relevance of manganese because its measured levels may reflect different aspects of exposure and metabolism depending on the matrix analyzed. Manganese plays a role in redox balance, cellular metabolism, and antioxidant defense, including mitochondrial superoxide dismutase activity [9,10,11]. However, these mechanisms were not directly assessed in the present study and should therefore be considered only as a potential biological background for the observed associations [12,13,14,15,22,46,47,48]. Obesity is characterized by chronic low-grade inflammation, mitochondrial dysfunction, and increased oxidative stress [49,50]. In this cardiometabolic context, altered manganese status may represent a marker of disrupted redox homeostasis rather than a direct determinant of atherosclerotic burden. Thus, the inverse relationship observed between hair manganese levels and BMI in our cohort may reflect redistribution or altered metabolism of this element under conditions of metabolic imbalance. However, this association was modest in magnitude and should be interpreted cautiously, as it may represent an exploratory finding rather than a robust clinical relationship. Experimental data indicate that excessive manganese accumulation may disrupt calcium homeostasis, nitric oxide signaling, and vascular tone regulation [51,52], whereas physiological levels support antioxidant defense mechanisms [19,20,21,53].

These findings should be interpreted in the context of previous studies examining the relationship between manganese intake and cardiovascular outcomes, which remain inconclusive [31,32,45]. For example, Nazeminezhad et al. reported that lower dietary manganese intake was associated with an increased risk of coronary artery disease [31], whereas Meishuo et al. suggested that higher manganese intake may reduce cardiovascular mortality [45]. However, both studies relied on food frequency questionnaires, which are prone to recall bias and do not directly reflect systemic manganese status. Estimated dietary intake may not correspond to biological availability, which depends on intestinal absorption, interactions with other trace elements—particularly iron—and tissue distribution. Similarly, Cebi et al. found no significant correlation between serum trace element levels, including manganese, and coronary artery disease [30], which is consistent with our results, although their study was limited by a small sample size. Evidence directly linking systemic manganese status with the angiographic severity of coronary artery disease remains limited. Consistent with our findings, Bagheri et al. reported no significant association between serum manganese levels and disease severity [35]. In contrast, data regarding acute clinical presentations are even more limited and inconsistent. Usui et al. suggested that serum Mn-SOD levels may reflect mitochondrial injury in the late phase of myocardial infarction [34], whereas Abdelrauf et al. demonstrated an association between a polymorphism reducing Mn-SOD activity and an increased risk of myocardial infarction [54]. However, these studies focused on enzymatic activity or genetic variability rather than systemic long-term manganese status, and the exclusion of patients with pre-existing cardiovascular disease limits the generalizability of their findings. The discrepancies between studies may be explained by several factors. First, different biological matrices used to assess manganese, including serum, urine, dietary intake, and hair, reflect distinct aspects of exposure and metabolism. Second, study populations differ with respect to clinical characteristics, metabolic burden, and environmental exposure. Third, manganese bioavailability is influenced by multiple factors, including intestinal absorption, tissue distribution, and interactions with other trace elements, particularly iron [19,20,21,22]. These findings suggest that hair manganese is not a reliable biomarker of angiographic coronary artery disease severity or ACS. This may be partly explained by the fact that hair reflects long-term metabolic status, whereas serum measurements are more sensitive to short-term changes associated with acute events. As noted above, BMI was the only clinical parameter nominally associated with hair manganese levels. This finding suggests that long-term manganese status may be more closely related to metabolic characteristics than to structural indicators of atherosclerosis, particularly in a cohort in which nearly 70% of patients were overweight or obese. Manganese is a cofactor for enzymes involved in carbohydrate and lipid metabolism, as well as oxidative stress regulation [7,9,55]. Population-based studies have also demonstrated associations between manganese intake and metabolic disorders, including metabolic syndrome and type 2 diabetes [56,57]. In addition, relationships between manganese status and oxidative stress markers across BMI categories have been reported, further supporting its potential role in metabolic regulation [55]. This may reflect altered metabolism, distribution, or demand for manganese under conditions of metabolic imbalance. Obesity and metabolic syndrome are associated with chronic low-grade inflammation, impaired redox homeostasis, and metabolic dysfunction [49,50,56,58], all of which may influence trace element metabolism. Our findings are partially consistent with those of Vahid et al. [59] and Aminnejad et al. [60], who reported inverse relationships between antioxidant-related dietary components and BMI, although their analyses were based on dietary data rather than biological measurements. It is also important to emphasize the biphasic biological profile of manganese: at physiological levels it participates in essential enzymatic and antioxidant processes, whereas excessive accumulation may contribute to redox imbalance and metabolic dysfunction [19,20,21,51,52,53]. Nevertheless, these mechanisms were not directly assessed in the present study. With regard to sex-related differences, no significant variations in Mn levels were observed in our cohort. Although some population-based studies have reported higher hair Mn concentrations in men compared with women, these differences may be influenced by environmental exposure patterns and methodological factors [61]. Experimental data also suggest that sex does not substantially affect Mn accumulation in tissues [62]. Therefore, our findings indicate that sex-related variability in manganese levels may be secondary to external or methodological factors rather than intrinsic biological differences.

Hypertension, present in nearly 90% of patients, was also analyzed in relation to Mn levels; however, no significant associations were observed. The relative homogeneity of the study population and the widespread use of antihypertensive therapy may have limited the ability to detect subtle relationships. Although oxidative stress and endothelial dysfunction are recognized contributors to hypertension pathophysiology [63,64,65], these mechanisms were not directly assessed in the present study and should be interpreted with caution. In addition, previously reported associations between Mn levels and blood pressure have been inconsistent, likely reflecting differences in study design, exposure assessment, and population characteristics. In our cohort with a high burden of established hypertension, potential modulatory effects of manganese may have been difficult to detect and could be more evident in lower-risk populations.

Type 2 diabetes, present in approximately one-third of participants, was not significantly associated with Mn status. Although manganese is involved in carbohydrate metabolism and insulin secretion [7,66], existing evidence is largely based on dietary assessments rather than direct biological measurements. The absence of a significant relationship in our study may reflect the complex and context-dependent nature of manganese metabolism in populations with advanced cardiovascular disease and multiple comorbidities.

Similarly, dyslipidemia was not associated with hair Mn levels. While manganese has been implicated in lipid metabolism [7], findings from both experimental and clinical studies remain inconsistent. Studies based on dietary intake have suggested inverse associations with lipid parameters [67]; however, such estimates do not necessarily reflect systemic manganese status. Overall, the heterogeneous findings in the literature indicate that the relationship between manganese and lipid metabolism is likely influenced by exposure level, biological matrix, and underlying metabolic context.

Importantly, the lack of significant associations between hair manganese levels and both angiographic severity of coronary atherosclerosis and the occurrence of ACS persisted after adjustment for major cardiometabolic confounders. This further supports the absence of an independent relationship between manganese status and structural coronary disease burden. Overall, our findings suggest that manganese is not a reliable biomarker of angiographic disease severity but may instead reflect metabolic characteristics within a cardiometabolic context. This distinction highlights the importance of interpreting trace element status not only in relation to vascular pathology but also within the broader framework of metabolic regulation.

## 5. Limitations

This study has several important limitations. First, the relatively small sample size, combined with the absence of a formal a priori sample size calculation, may have reduced statistical power, particularly in analyses of ACS subtypes, angiographic severity, and weaker associations. As this was an exploratory observational study based on consecutively enrolled eligible patients with complete clinical and laboratory data, the findings should be interpreted with caution and considered hypothesis-generating, pending confirmation in larger prospective cohorts.

Second, the cross-sectional design precludes causal inference. Additionally, the retrospective design and the time frame of data collection (2013–2017) should be considered when interpreting the findings. Changes in clinical practice, including advances in pharmacotherapy and revascularization strategies, as well as potential shifts in population characteristics over time, may limit the generalizability of the results to contemporary clinical settings. Third, manganese status was assessed using a single biological matrix, namely hair. Although hair is a useful indicator of long-term exposure, it does not provide a comprehensive assessment of current metabolic status or manganese distribution across body compartments. Furthermore, external contamination of hair samples cannot be entirely excluded. Although standardized washing and preparation procedures were applied prior to analysis, environmental exposure (e.g., dust, water, or cosmetic products) may have influenced manganese measurements, which is an inherent limitation of hair-based biomarker assessment. Fourth, dietary manganese intake and nutritional factors influencing its absorption were not assessed in this study. Manganese bioavailability is strongly modulated by diet composition and interactions with other trace elements, particularly iron, which shares common transport pathways. Iron deficiency may increase manganese absorption, whereas adequate or high iron status may reduce its uptake. The absence of data on dietary manganese intake, iron status, and broader micronutrient interactions limits the interpretation of the observed associations. In addition, the potential impact of pharmacological treatment was not analyzed in detail. Medications commonly used in this population, including statins, antihypertensive agents, and antidiabetic drugs, may influence oxidative stress, inflammatory pathways, and trace element metabolism. Therefore, their potential confounding effect on manganese status cannot be excluded.

Finally, although an association between manganese levels and BMI was observed, this finding should be interpreted cautiously as exploratory and hypothesis-generating. The study did not include direct evaluation of molecular or functional markers of oxidative stress and mitochondrial metabolism; therefore, the biological interpretation of this finding should be made with caution [12,13,14,15,22,46,47,48].

Additionally, the relatively large number of statistical comparisons increases the risk of type I error, even though appropriate statistical corrections were applied. Therefore, nominally significant findings should be interpreted with caution.

## 6. Conclusions

This study provides evidence that hair manganese concentration reflects long-term manganese exposure in patients with coronary artery disease. Although no significant association was observed between hair manganese levels and angiographic severity or the occurrence of ACS, the inverse relationship with BMI—although modest—suggests a potential exploratory link between manganese homeostasis and metabolic regulation in a cardiometabolic context. Given the dual biological role of manganese as an essential micronutrient that may exert pro-oxidative effects at higher concentrations, maintaining its levels within a narrow physiological range appears important for redox balance and metabolic stability. Rather than directly reflecting structural atherosclerotic burden, manganese status may indicate broader interactions among trace element homeostasis, oxidative stress, and metabolic phenotype. Future studies incorporating environmental exposure assessment, multiple biological matrices, and more detailed clinical characterization are needed to better define the role of manganese in cardiovascular and metabolic health.

## Figures and Tables

**Table 1:** Differences in selected parameters between patients with different diagnoses.

Variable	Stable CAD	STEMI	NSTEMI	UA	*p*-Value
Number (♀/♂)	15/27	2/15	3/9	3/6	0.313
Age (years)	67 ± 10	64 ± 15	70 ± 8	72 ± 8	0.236
BMI (kg/m^2^)	27 (21–41)	27 (17–30)	27 (23–45)	29 (23–36)	0.437
Smoking (active/former smoker/no)	9/7/26	8/0/9	3/0/9	2/0/7	0.115
Hypertension (yes/no)	36/6	14/3	11/1	9/0	0.570
t2DM (yes/pre-diabetes/no)	15/3/24	5/1/11	1/2/9	4/0/5	0.487
TC (mg/dL)	159.5 (96.4–256.7)	215.9 (114.1–293.6)	159.6 (70.0–211.7)	174.0 (107.9–310.8)	0.008
HDL (mg/dL)	48.5 (18.6–97.4)	48.0 (30.2–67.7)	47.1 (29.5–54.6)	47.1 (33.5–66.5)	0.547
LDL (mg/dL)	78.6 (26.2–172.9)	135.4 (49.5–213.6)	91.6 (31.8–130.3)	106.4 (48.1–228.3)	0.003
TG (mg/dL)	121.0 (66.6–357.6)	108.8 (68.2–367.8)	96.7 (43.5–157.1)	94.9 (69.2–189.0)	0.299
Hyperlipidemia (yes/no)	17/23	10/6	2/7	4/5	0.265
Previous MI (yes/no)	15/27	5/12	4/8	2/7	0.871
CASSS (0/1/2/3)	12/9/14/7	0/8/5/4	1/3/3/5	2/1/4/2	0.148
Syntax (points)	8.0 (0.0–58.0)	18.0 (3.0–64.5)	23.8 (0.0–68.5)	14.0 (0.0–36.0)	0.027
Mn (ppm)	0.24 (0.03–1.65)	0.29 (0.07–1.33)	0.12 (0.01–0.66)	0.18 (0.01–1.30)	0.181

Data presented as number (%) mean ± SD or median (range). BMI—body mass index; t2DM—type 2 Diabetes Mellitus; TC—total cholesterol; HDL—high-density lipoprotein cholesterol; LDL—low-density lipoprotein cholesterol; TG—triglycerides; MI—myocardial infarction; CASSS—Coronary Artery Surgery Study Score; Mn—manganese; CAD—coronary artery disease; STEMI—ST-elevation myocardial infarction; NSTEMI—non-ST-elevation myocardial infarction; UA—unstable angina.

**Table 2:** Association between selected parameters, including Mn level and CAD stages.

	CASSS 0	CASSS 1	CASSS 2	CASSS 3	*p*-Value
Number (♀/♂)	8/7	8/13	3/23	4/14	0.024
Age (years)	69 ± 11	69 ± 12	64 ± 11	69 ± 9	0.565
BMI (kg/m^2^)	28 (21–36)	27 (17–41)	28 (23–36)	26 (19–45)	0.761
Smoking (active/former smoker/no)	3/0/12	5/1/15	11/5/10	3/1/14	0.054
Hypertension (yes/no)	11/4	18/3	24/2	17/1	0.245
t2DM (yes/pre-diabetes/no)	2/1/12	8/0/13	8/3/15	7/2/9	0.429
TC (mg/dL)	180.5 (134.0–255.9)	166.9 (101.7–256.7)	159.9 (98.7–298.5)	144.5 (70.0–310.8)	0.230
HDL (mg/dL)	47.5 (28.6–66.5)	49.6 (30.2–97.4)	47.8 (32.4–66.5)	44.7 (18.6–60.4)	0.278
LDL (mg/dL)	111.7 (65.9–171.7)	100.4 (26.2–165.6)	88.7 (26.9–213.6)	76.8 (31.8–228.3)	0.428
TG (mg/dL)	128.3 (94.3–281.0)	97.5 (66.7–357.4)	115.1 (61.9–367.8)	113.4 (43.5–189.0)	0.189
Hyperlipidemia (yes/no)	7/7	10/11	11/13	5/10	0.795
Previous MI (yes/no)	0/15	6/15	11/15	9/9	0.012
Syntax (points)	0.0 (0.0–38.0)	8.0 (0.0–30.0)	18.5 (4.0–48.0)	32.8 (6.0–68.5)	<0.001
Mn (ppm)	0.24 (0.09–1.65)	0.21 (0.01–1.33)	0.23 (0.03–1.30)	0.19 (0.01–1.00)	0.819

Data presented as number (%) mean ± SD or median (range). BMI—body mass index; t2DM—type 2 Diabetes Mellitus; TC—total cholesterol; HDL—high-density lipoprotein cholesterol; LDL—low-density lipoprotein cholesterol; TG—triglycerides; MI—myocardial infarction; Mn—manganese; CASSS—Coronary Artery Surgery Study Score.

**Table 3:** Associations between Mn concentration and selected clinical variables.

Variable	Estimate	95% CI	*p* Value	Adjusted *p* Value FDR	Adjusted *p* Value Bonferroni
Age	−0.114	−0.325 to 0.109	0.316	0.487	1.000
BMI	−0.250	−0.450 to −0.026	0.029	0.262	0.263
TC	0.200	−0.029 to 0.410	0.087	0.262	0.784
HDL	0.105	−0.129 to 0.327	0.379	0.487	1.000
LDL	0.180	−0.052 to 0.394	0.127	0.287	1.000
TG	0.041	−0.189 to 0.267	0.728	0.728	1.000
SYNTAX	−0.106	−0.319 to 0.116	0.348	0.487	1.000

BMI—body mass index; TC—total cholesterol; HDL—high-density lipoprotein cholesterol; LDL—low-density lipoprotein cholesterol; TG—triglycerides.

## Data Availability

Data can be provided by the corresponding author upon reasonable request due to restrictions related to privacy, legal, or ethical considerations.

## Supplementary Materials

The following supporting information can be downloaded at https://www.mdpi.com/article/10.3390/nu18071089/s1. Figure S1: Conceptual model of manganese (Mn) dyshomeostasis in cardiometabolic regulation. The schematic presents a proposed framework linking Mn homeostasis with redox balance and metabolic stress. Physiologically, Mn functions as a cofactor of mitochondrial superoxide dis-mutase (MnSOD), supporting antioxidant defense. In conditions of metabolic stress (e.g., elevated BMI), altered Mn availability and reduced SIRT3 activity may impair MnSOD function, leading to increased mitochondrial ROS, inflammatory signaling (NF-κB), endothelial dysfunction, and profibrotic activation (TGF-β/Smad). This model is based on previously published data and does not reflect direct mechanistic measurements performed in the present study.

## Abbreviations

The following abbreviations are used in this manuscript:

ACS	Acute coronary syndrome
BMI	Body mass index
CAD	Coronary artery disease
CASSS	Coronary Artery Surgery Study Score
CCS	Chronic coronary syndrome
CVDs	Cardiovascular diseases
EAS	European Atherosclerosis Society
ECG	Electrocardiogram
ESC	European Society of Cardiology
FFQ	Food Frequency Questionnaire
HDL	High-density lipoprotein cholesterol
H_2_O_2_	Hydrogen peroxide
ICP-OES	Inductively coupled plasma optical emission spectrometry
ICAM-1	Intercellular Adhesion Molecule 1
LAD	Left anterior descending artery
LCX	Left circumflex artery
LDL	Low-density lipoprotein cholesterol
MI	Myocardial Infarction
Mn	Manganese
MnSOD	Manganese superoxide dismutase
NF-κB	Nuclear Factor kappa-light-chain-enhancer of activated B cells
NSTEMI	Non-ST-elevation myocardial infarction
OGTT	Oral glucose tolerance test
O_2_•^−^	Superoxide radicals
PCI	Percutaneous coronary intervention
RCA	Right coronary artery
R	Spearman correlation coefficient
ROS	Reactive oxygen species
SIRT3	Sirtuin 3
Smad 2	SMAD Family Member 2
Smad 3	SMAD Family Member 3
SNP	Single Nucleotide Polymorphism
STEMI	ST-elevation myocardial infarction
TC	Total cholesterol
TG	Triglycerides
TGF-β	Transforming Growth Factor beta
t2DM	Type 2 Diabetes Mellitus
UA	Unstable angina
WHO	The World Health Organization
