# Supplementary material for: Hair Manganese as a Marker of Cardiometabolic Status Rather than Coronary Artery Disease Severity—An Exploratory Pilot Study
Source: Nutrients. 2026 Mar 28;18(7):1089. doi: 10.3390/nu18071089 (PMC13074630; doi:10.3390/nu18071089)
Supplement: Supplementary file 1 [file nutrients-18-01089-s001.zip › nutrients-4198315-supplementary.pdf]

Supplementary Materials:

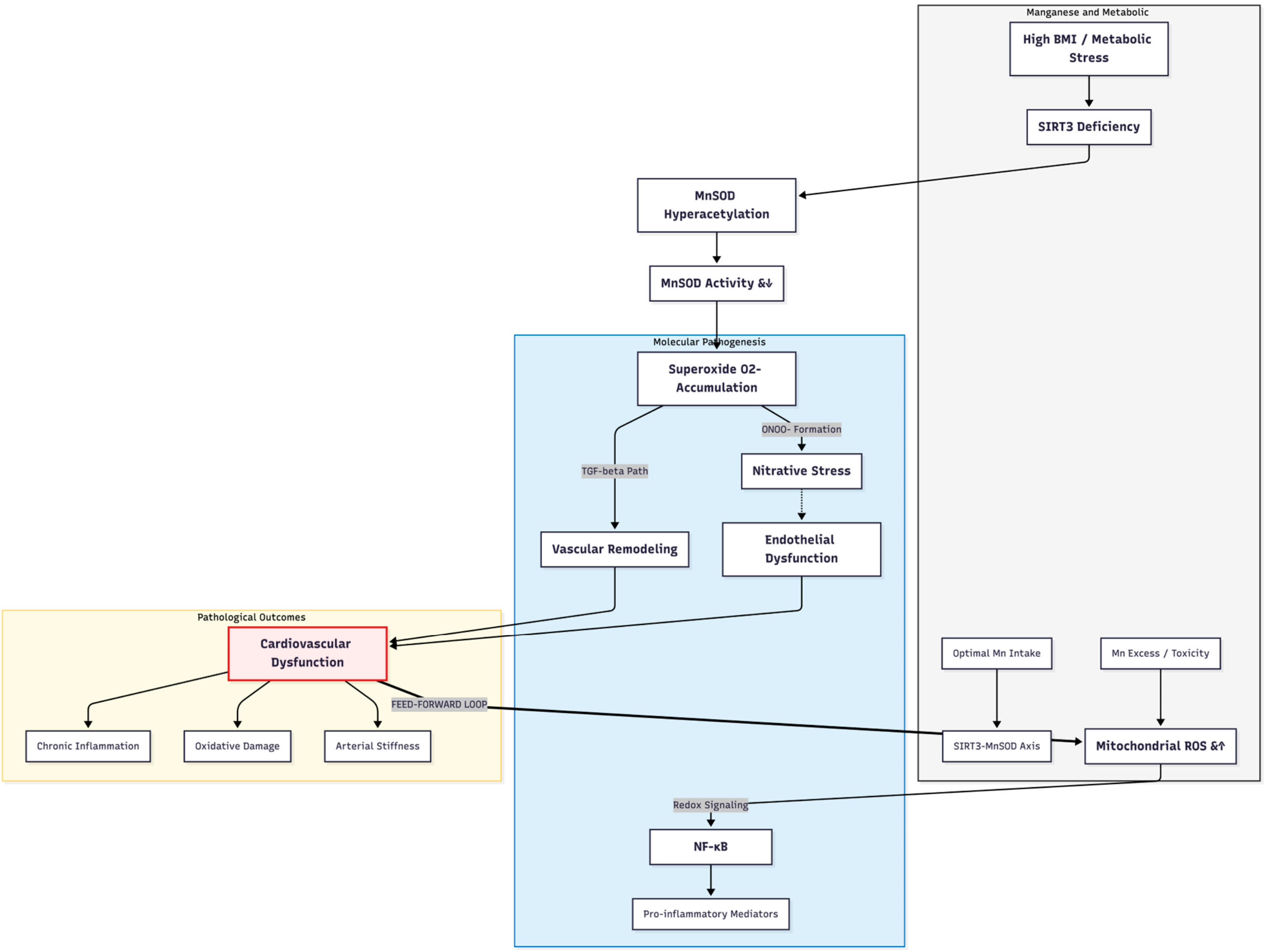

Supplementary Figure S1. Conceptual model of manganese (Mn) dyshomeostasis in cardiometabolic regulation. The schematic presents a proposed framework linking Mn homeostasis with redox balance and metabolic stress. Physiologically, Mn functions as a cofactor of mitochondrial superoxide dismutase (MnSOD), supporting antioxidant defense. In conditions of metabolic stress (e.g., elevated BMI), altered Mn availability and reduced SIRT3 activity may impair MnSOD function, leading to increased mitochondrial ROS, inflammatory signaling (NF-κB), endothelial dysfunction, and profibrotic activation (TGF-β/Smad). This model is based on previously published data and does not reflect direct mechanistic measurements performed in the present study.
